# Engineering of Phage-Derived Lytic Enzymes: Improving Their Potential as Antimicrobials

**DOI:** 10.3390/antibiotics7020029

**Published:** 2018-03-22

**Authors:** Carlos São-José

**Affiliations:** Research Institute for Medicines (iMed.ULisboa), Faculty of Pharmacy, Universidade de Lisboa, Av. Prof. Gama Pinto, 1649-003 Lisboa, Portugal; csaojose@ff.ul.pt; Tel.: +351-217-946-420

**Keywords:** endolysin, lysin, lytic enzyme, peptidoglycan hydrolase, antimicrobial, antibacterial, antibiotic resistance, antimicrobial resistance, bacteriophage

## Abstract

Lytic enzymes encoded by bacteriophages have been intensively explored as alternative agents for combating bacterial pathogens in different contexts. The antibacterial character of these enzymes (enzybiotics) results from their degrading activity towards peptidoglycan, an essential component of the bacterial cell wall. In fact, phage lytic products have the capacity to kill target bacteria when added exogenously in the form of recombinant proteins. However, there is also growing recognition that the natural bactericidal activity of these agents can, and sometimes needs to be, substantially improved through manipulation of their functional domains or by equipping them with new functions. In addition, often, native lytic proteins exhibit features that restrict their applicability as effective antibacterials, such as poor solubility or reduced stability. Here, I present an overview of the engineering approaches that can be followed not only to overcome these and other restrictions, but also to generate completely new antibacterial agents with significantly enhanced characteristics. As conventional antibiotics are running short, the remarkable progress in this field opens up the possibility of tailoring efficient enzybiotics to tackle the most menacing bacterial infections.

## 1. Introduction

Alexander Fleming himself was the first to recognize that bacteria could easily develop resistance to penicillin after prolonged exposure to the antibiotic. Since their introduction in clinical practice in the 1940s, antibiotics have been overused and misused both in humans and animals. Over the years this led to the uncontrolled emergence and spread of antibiotic-resistant determinants in almost all bacterial pathogens, some of which are becoming highly refractory to all current antibiotics [1]. As a consequence, we risk entering a post-antibiotic era where we will no longer be able to efficiently treat common bacterial infections [2]. Several international studies anticipate truly catastrophic scenarios on a global scale if effective solutions to tackle antimicrobial resistance are not rapidly found, with tens of million deaths per year and costs ascending to trillions of USD by 2050 [3,4]. This threat, associated with a very limited pipeline of truly new therapies from the pharmaceutical industry [5], has been driving research on alternative antimicrobials, preferentially on those with new modes of action to minimize resistance development. Among the most promising alternatives or complements to conventional antibiotics are phage-derived lytic enzymes [6]. Other phage-encoded enzymes with potential applications as antibacterial weapons will not be discussed in this review. These include, for example, the polysaccharide depolymerases that degrade bacterial capsules, biofilms, and the outer membrane lipopolysaccharide of Gram-negative bacteria [7,8,9,10].

Phage lytic enzymes (PLEs) harbor at least one domain responsible for the enzymatic cleavage of peptidoglycan, also known as murein, which is the major structural component of the bacterial cell wall (CW). The peptidoglycan macromolecule forms a sacculus that surrounds the bacterial cytoplasmic membrane (CM) and confers the necessary mechanical resistance to avoid cell lysis as a result of turgor pressure [11]. Therefore, uncontrolled breakdown of the murein structure typically results in osmotic cell lysis. The vast majority of known phages (tailed phages) need to breach the rigid CW barrier in two essential steps of the infection cycle; first, to deliver the virus genetic material into host cells, and then to allow escape of the virion progeny from infected cells. The PLEs that participate in the entry step attack the CW from the outside of the cell (from without). They are transported in the virus particle and can therefore be called virion-associated lysins (VALs) [10,12]. Those responsible for virion release after virus multiplication are synthesized in the host cell during the course of infection. At the appropriate time for lysis to occur, they attack the CW from within and have thus been called endolysins [13]. Although in the literature the term endolysin is very frequently shortened to lysin, this simplification will not be used in this review to avoid confusion. Likewise, VALs are also known as virion-associated peptidoglycan hydrolases (VAPGH), tail-associated muralytic enzymes (TAME), tail-associated lysins (TAL), exolysins, or structural lysins. As argued by Lakta et al. [10] though, virion-associated lysin is probably the best suited designation and will be the one adopted here.

In the natural context of phage infection, the CW peptidoglycan is cleaved by PLEs in a controlled manner, first to avoid compromising host cell viability during phage DNA delivery and then to make sure that lysis does not occur before completion of the replicative cycle (see Section 2 for details). However, when added exogenously to bacteria in the form of purified proteins, the murein-degrading activity of PLEs can lead to rapid osmotic lysis and consequently to cell death. This property has been the basis for the exploration of PLEs as antibacterial agents, as part of a broad group of lytic enzymes defined as “enzybiotics” [14]. The therapeutic potential of endolysins as antibacterial agents has been intensively studied for about twenty years now, both in vitro and in animal models of infection/colonization, with very promising results (for reviews see [15,16,17,18]). Since the outer membrane of Gram-negative bacteria most frequently blocks the access of lytic enzymes added from without to the murein layer, the initial stages of the enzybiotics field relied mainly on endolysins targeting Gram-positive pathogens. Recent developments, however, have opened venues for great expansion of the field to Gram-negative pathogens (Section 5.5). Interest in VALs as enzybiotics emerged more recently [10,19], but, as discussed below, they exhibit properties that may confer them some advantages when compared to endolysins. Besides clinical use, PLEs may also find pathogen control applications in other fields such as diagnostics, food industry, and agriculture [20,21,22].

When employed as antibacterial agents, both VALs and endolysins will have to exert their lytic activity in conditions substantially different from those found in their native context of action. These “unnatural” conditions may negatively impact the performance of these lytic agents. In fact, the activity of VALs at the initial stages of phage infection is most often assisted by other virion components and by dynamic rearrangements of the virus structure. On the other hand, endolysins are naturally designed to attack the CW from within and after the action of a second phage-encoded lysis product: the holin (see below). In a therapeutic perspective, recombinant PLEs will also have to display their killing activity in complex environments such as animal tissues, mucosal membranes, blood and body fluids. In addition to the optimization of killing efficiency, it may be necessary to modify PLEs to ameliorate other properties such as their spectrum of activity, solubility, stability, and half-life in infected hosts. Moreover, the proteinaceous nature of PLEs and their capacity to induce lysis of target bacteria is expected to generate host immune responses, with possible adverse effects, along with the production of antibodies that might neutralize the therapeutic agents. Interestingly, the available studies addressing these potential problems, including several safety studies in humans, reported no serious adverse effects and low impact of anti-PLE antibodies in antimicrobial activity (for a review of immunity and safety issues, and of endolysins in clinical stages, see [23]). Nevertheless, host immune responses must always be considered, and some PLEs may require intervention to improve their therapeutic potential in this regard.

Based on several examples, the following sections will present how PLE features can be improved through protein modification and engineering strategies. Since the rational for several of the PLE tailoring approaches resulted from key knowledge on the mode of action and biochemical characteristics of the lytic enzymes, these aspects we will be covered first.

## 2. Mode of Action of VALs and Endolysins during Phage Infection

**Figure 1:**
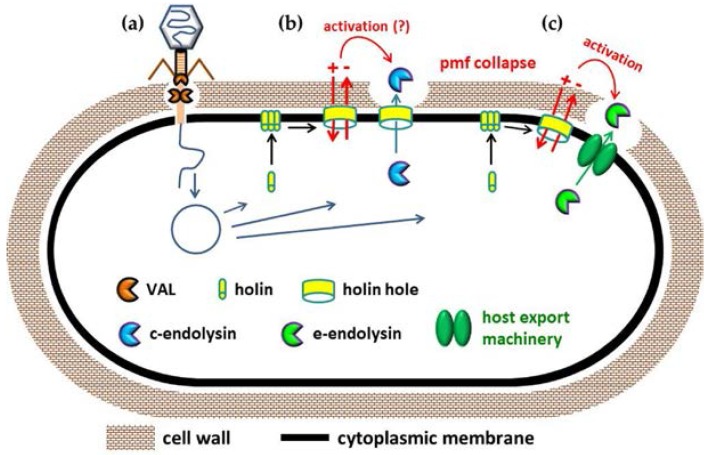
Natural context of action of phage lytic enzymes (PLEs). (**a**) Virion-associated lysins (VALs) promote a local digestion of the cell wall (CW) peptidoglycan to assist penetration of the phage tail tube and passage of the viral DNA to the host cell cytoplasm. After phage genome expression, infected cells must lyse to release the newly-formed virus particles. This is achieved thanks to the peptidoglycan-degrading activity of endolysins; (**b**) Most known endolysins gain access to the CW compartment through the holin channels (c-endolysins); (**c**) Some, however, are exported (e-endolysins) via host cell machineries (e.g., the bacterial Sec system). Holin-mediated dissipation of the cytoplasmic membrane proton-motive force (pmf) is an essential requirement for activation of e-endolysins, while it may also potentiate the lytic activity of c-endolysins (see text).

Understanding the biological context and mode of action of PLEs may provide valuable information for their exploration as enzybiotics. VALs are typically associated with the phage DNA injection machinery. Most frequently, they correspond to individual components or to domains of phage tail substructures, like the tape measure protein, central fibers, tail tip knobs, and tail tip puncturing devices, but they can also be capsid inner proteins [10,12,19]. Specific binding of phage tail receptor binding proteins (RBPs) to host cell surface receptors triggers major virion conformational changes that place VALs in close contact with the bacterial CW [24]. This frequently involves the protrusion and insertion in the bacterial cell envelope of tail structures that carry VALs. A landmark example is provided by myoviruses like phage T4 and their contractile tails. Upon tail sheath contraction, the tail tube with a puncturing device at its tip penetrates the cell envelope [25]. One of the proteins composing the piercing apparatus in T4 is gp5, which has muralytic activity [26,27]. Some siphoviruses (long, non-contractile tails) were shown to eject and insert in the cell envelope the internal tail tube formed by the tape measure protein, which may also be endowed with peptidoglycan-cleaving activity [28,29]. After irreversible binding to cell receptors, podoviruses (short tails) like phage T7 eject the proteins composing the capsid inner core to form an extended tail tube that spans the cell envelope. One of the T7 core proteins is gp16 that has CW-degrading activity ([30] and references therein). Other podoviruses like φ29 simply seem to drill through the CW, helped by the peptidoglycan hydrolysis activity of one of the components of the tail tip knob [31]. In conclusion, independently of the mechanism of action, VALs are thought to promote a local digestion of the peptidoglycan structure to allow penetration or extension of the tail tube across the CW and its subsequent fusion to the CM. Tail tube insertion in the CM is then followed by the translocation of the viral DNA into the bacterial cytoplasm [32] (Figure 1a).

Endolysins are produced in the cytoplasm of infected cells and need, therefore, to overcome the CM barrier in order to reach the CW and exert their lytic action. These enzymes can be generally classified in canonical endolysins (c-endolysins) or exported endolysins (e-endolysins), depending on the pathway they follow to reach the CW. In the first case, endolysins require the action of a second phage-encoded function, the holin, to be able to escape the cytoplasm. Holins build and oligomerize in the CM, and at a genetically programed time they are triggered to form holes, which cause the lethal collapse of the CM proton-motive force (pmf) [33]. Additionally, these holes are large enough to allow escape of the cytoplasm-accumulated c-endolysin to the CW, which is an essential requirement for occurring lysis. This mechanism defines the so-called canonical lysis model and is prototyped by *Escherichia coli* phage λ [33] (Figure 1b). The endolysins that have been explored as enzybiotics are c-endolysins.

The currently known non-canonical lysis systems employ e-endolysins [13,34,35]. Here, the lytic enzymes are exported to the CW compartment by engaging host cell transport machineries, most frequently the general secretion pathway (the Sec system). E-endolysins start to accumulate in the CW during the viral reproductive cycle, but their lytic activity is inhibited by mechanisms that depend on an energized CM. In fact, although holins do not participate in endolysin channelling to the CW, they still maintain the key role of defining the lysis timing thanks to their pmf-dissipating action, which abolishes the mechanisms that restrain the lytic enzymes (Figure 1c). Interestingly, it was recently shown that energized bacterial cells can also counteract the lytic action of c-endolysins when artificially exported to the CW (from within), or when added from without as recombinant proteins. These results suggest that in canonical lysis, holins may also “activate” endolysins as result of their pmf-collapsing activity [36].

A notion that easily emerges from the comparison of the mode of action of the two types of PLEs is that VALs are “naturally designed” to act on the CW of viable and dividing bacteria, whereas in their natural context both c- and e-endolysins cut the peptidoglycan of cells that are first killed by the holin.

## 3. Enzymatic Activity of PLEs

**Figure 2:**
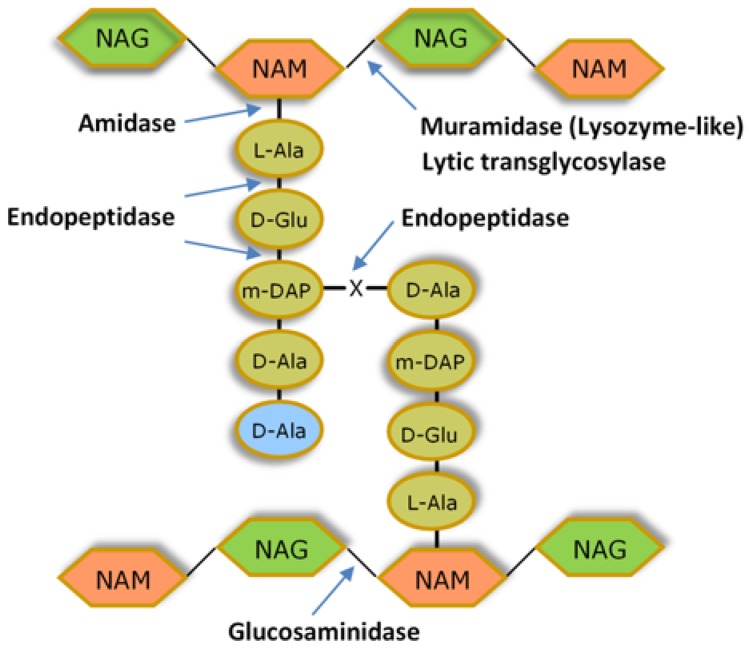
Basic structure of the bacterial cell wall peptidoglycan. The possible enzymatic activities of PLEs and the bonds they cleave are indicated. Typically, PLEs carry one or two catalytic domains displaying one of the indicated enzymatic activities. m-DAP is found in the peptide chains of the peptidoglycan of most Gram-negative bacteria, *Bacillus* spp. and *Listeria* spp., which present also direct m-DAP-D-Ala bonding between adjacent stem peptides. In most Gram-positive bacteria, m-DAP is replaced by L-Lys. Cross-linking between this residue and D-Ala of a neighbor peptide chain usually occurs by an interpeptide bridge of variable amino acidic composition (X). Despite some variation observed among isolates of the same bacterial species, examples of X bridges are (Gly)_5_ found in *Staphylococcus aureus*, L-Ala-L-Ala in *Enterococcus faecalis* and *Streptococcus pyogenes*, D-Asp in *E. faecium*, and L-Ser-L-Ala in *S. pneumoniae*. The D-Ala residue in light blue may be lost after peptidoglycan maturation.

The antibacterial potential of PLEs resides in their capacity to cleave different bonds of the CW peptidoglycan network. The murein polymer has as repeating unit a disaccharide made of *N*-acetylglucosamine (NAG) and *N*-acetylmuramic acid (NAM), linked by glycosidic bonds *β* (1 → 4). Neighbouring glycan strands are cross-linked by penta/tetrapeptide side stems that are attached to NAM via amide bonds. The most frequent type of cross-linking involves the amino acid residues at positions 3 (often L-Lys or meso-diaminopimelic acid (m-DAP)) and 4 (D-Ala) of adjacent peptide chains. The peptide stems are interconnected by a direct interpeptide bond in most Gram-negative bacteria and few Gram-positive species, whereas peptide chain cross-linking occurs via an interpeptide bridge in the majority of Gram-positive bacteria. The different peptidoglycan types among bacteria derive mostly from variations within the peptide moiety, notably in the amino acidic composition of the interpeptide bridges [11,37] (Figure 2).

The catalytic domains (CDs) of PLEs, also referred to as enzymatically active domains (EADs), can be classified into three major categories according to their murein cleavage specificity: glycosidases, amidases, and endopeptidases [10,15]. Glycosidases cleave one of the two glycosidic bonds in the glycan chain and can be subdivided into *N*-acetyl-*β-*d-glucosaminidases (glucosaminidases), *N*-acetyl-*β-*d-muramidases (muramidases or lysozymes), and lytic transglycosylases (Figure 2). Amidases (*N*-acetylmuramoyl-l-alanine amidases) hydrolyse the amide bond connecting NAM to the first amino acid residue of the peptide stem (generally L-Ala), while endopeptidases cleave within or between the peptide strands (Figure 2). All CDs seem to break the peptidoglycan though a hydrolysis mechanism, except lytic transglycosylases [15].

The most common CDs in known endolysins specify muramidase or amidase activity, whereas VALs seem to carry preferentially glycosidase and endopeptidase activities (the latter almost exclusively present in phages infecting Gram-positive bacteria). The CD may impact the range of activity of a given PLE, depending on whether it targets widely conserved peptidoglycan bonds (such as the glycosidic bonds) or linkages that are specific to particular CW types (like the pentaglycine peptide bridge of staphylococcal CW).

The increasing number of PLE sequences deposited in protein databases has enabled the organization of CDs and CW binding domains (see next) into different families and superfamilies [15,38]. This classification coupled to powerful bioinformatics tools [39] frequently allow the inclusion of PLE CDs into known superfamilies/families and infer about the type of muralytic activity. This analysis, however, should be confirmed experimentally to avoid erroneous assumptions. For example, CDs of the CHAP family most often display endopeptidase activity [40,41], but they can also work as amidases [42] or even exhibit both types of activity [43]. Another interesting observation is that CDs presenting the same murein cleavage specificity can be grouped into distinct families according to their sequence relatedness.

## 4. Domain Architecture of PLEs

In their simplest form, PLEs are monomeric, globular proteins that essentially correspond to the CD responsible for cleaving a specific peptidoglycan bond. In fact, this simple structure is predominant among endolysins of phages infecting Gram-negative bacteria. In the next step of complexity, endolysins may possess a cell wall binding domain (CWBD or CBD) connected to the CD by a linker segment. The CWBD has high affinity to a particular cell envelope component, and, in general, it contributes positively to the lytic action of endolysins against natural target bacteria. According to the level of conservation of the cell envelope ligand, the CWBD will variably influence the endolysin spectrum of activity. It should be noted, however, that depending on the endolysin and/or target bacteria, the presence of CWBD may or may not be essential for enzybiotic applications (see Section 5.2). Well-known examples of CW binding motifs targeting the murein or other polymeric components of the CW include LysM, SH3 (with different subtypes), the cell binding domains (CBDs) of listerial endolysins, and the choline-binding repeats (ChBRs) of streptococcal endolysins [44,45,46,47]. Modular endolysins with an N-terminal CD region coupled to a C-terminal CWBD are typical of phages infecting Gram-positive hosts and mycobacteria [15,48]. The modular character of endolysin CDs and CWBDs was early recognized in pioneer studies of the lytic enzymes of *S. pneumoniae* and its phages, which showed that natural chimeric enzymes are generated through evolution by the interchange of cleavage and binding modules [47,49].

**Figure 3:**
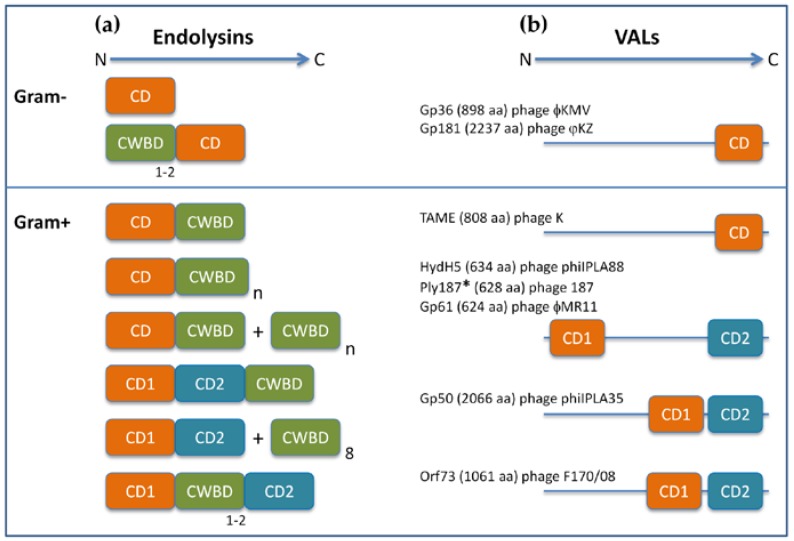
Domain architecture of endolysins (**a**) and VALs (**b**) that have been explored as enzybiotics, from Gram-positive and Gram-negative systems. CD, catalytic domain; CWBD, cell wall binding domain. The cardinals indicate the copy number of CW binding motifs composing the CWBD. The “n” letter indicates that a variable number of CW binding motifs may compose the CWBD (2 to 7 copies). These may be present either as tandem repetitions (in monomeric enzymes) or as oligomers when the CWBD subunit is independently produced by in-frame, alternative start sites (see text). The subunits of hetero-oligomeric endolysins are separated by the “+” sign. The presented VALs are from phages infecting *Pseudomonas aeruginosa* [56,57], *S. aureus* [58,59,60,61], and *E. faecalis* [62]. * Ply187 was firstly described as an endolysin. Schemes of phage lytic enzymes are not drawn to scale.

In addition to the two basic domain configurations referred to above, other endolysin architectures exist that essentially vary regarding the presence, number, and relative position of CDs and CWBDs (for reviews on the diversity of endolysin domain architectures see [15,38,50]). For example, endolysins equipped with two CDs that target distinct peptidoglycan bonds or that carry tandem repetitions of CW binding motifs are quite common in Gram-positive systems. Moreover, a considerable number of endolysins with peptidoglycan binding modules at their N-terminus have already been described for phages infecting Gram-negative hosts ([51,52] and references therein). It is also worth noting that some endolysins seem to work as hetero-oligomeres, in which CD-containing subunits associate with several independently produced copies of the CWBD. In the case of the streptococcal multimeric endolysin PlyC, the dual-CD subunit (PlyCA) and the 8-mer CWBD (PlyCB) are expressed from separate genes [42,53]. In the case of the enterococcal Lys170 and of the clostridial CD27L and CPT1L lytic enzymes, a single gene with an internal translation start site generates both the full-length endolysin and independent CWBD subunits, which then associate [54,55]. Figure 3a illustrates the CD and CWBD arrangements found among endolysins that have been studied as antibacterial agents. Besides CD and CWBD modules, most known endolysins involved in non-canonical lysis modes (e-endolysins, see Section 2) are endowed with N-terminal secretion signals such as Sec-type signal peptides or “signal-anchor-release” (SAR) domains [13,35].

VALs are much less studied than endolysins regarding their biochemical properties, murein degrading activity, and antimicrobial potential. In addition to the function associated with murein cleavage, they may play a role in the morphogenesis and stability of phage virions. VALs tend to be multidomain and much larger than endolysins, and some are found as oligomers in the virus particle. Still, the VAL CDs responsible for cutting peptidoglycan are related to their counterparts in endolysins. In contrast to VALs of phages infecting Gram-negative bacteria, those of Gram-positive systems frequently carry two CDs, with distinct cleavage specificities, and with variable modular organization. The vast majority of known VALs do not exhibit any obvious CWBD under in silico analysis. The presence of this domain is probably dispensable, because VAL/CW contact is guaranteed by the tight interaction between phage tails and host cell surface receptors. The absence of CWBD in VALs constitutes a major difference relative to endolysins (for reviews on VALs features, see [10,19]). Figure 3b presents the domain architecture of some VALs that have been explored (or their CDs) as enzybiotics.

## 5. Improving the Potential of PLEs as Antibacterials through Protein Engineering

As referred earlier, over the past twenty years numerous studies have supported that native PLEs, particularly endolysins targeting Gram-positive bacteria, are capable of antibacterial activity when added from without. More recently, however, a growing number of reports have described enzybiotics with improved features as result of PLEs modification and engineering of new derivatives. Among the upgraded properties, we can find the enhancement of killing activity against bacteria growing in different conditions and environments, the expansion of the natural bactericidal spectrum, and the extension of PLEs as efficient antibacterial agents towards Gram-negative pathogens. At the production level, different strategies have been followed to increase enzybiotic solubility when heterologously expressed (almost always in *E. coli*) and subsequent stability. Remarkably, as we will see next, several of these improvements may be achieved by a single engineering approach.

The seminal studies demonstrating the modular character of the functional domains of peptidoglycan hydrolases from Gram-positive systems were the basis for the most widely used engineering strategy applied to PLEs, that is, the shuffling and fusion of CDs and CWBDs of different origin to generate chimeric lytic enzymes (also known as chimeolysins). The design of chimeolysins has been much helped by the increasing availability of PLEs tridimensional structures and bioinformatics tools. These normally allow identification and delimitation of enzyme functional domains at the sequence level, and fairly good prediction of their biochemical properties. The native features of PLEs functional domains are usually preserved after being combined to generate certain chimeolysins. This has been explored to tailor enzybiotics with specific properties.

Other tailoring approaches applied to PLEs include fusion of full-length lytic enzymes, domain deletion, addition or duplication, random or site-directed mutagenesis, fusion to peptides, and the combination of few of these [17,23,63]. Examples of PLE engineering or modification strategies and how they enhance specific enzybiotic features are provided next.

### 5.1. Generation of Chimeolysins with Increased Lytic Spectrum and Activity

The combination of PLEs functional domains of heterologous origin has been extensively used to produce chimeric enzymes with altered properties. Table 1 lists key examples of chimeolysins that clearly exhibited improvements when compared to their parental PLEs or to other lytic enzymes. When a CW binding module recognizes a ligand that is specific to a certain CW type, it might be expected that its fusion to a heterologous CD will retarget the activity of the latter, as long as the peptidoglycan bond matching the CD specificity is available in the new CW [64]. This was well demonstrated in a work with two *Listeria monocytogenes* phage endolysins, Ply118 and PlyPSA, in which individual CD and CWBD modules were swapped or combined to generate fusions with improved capacity to label and lyse *Listeria* cells [65]. Interestingly, one of the fusions (EAD118_III_CBDPSA) showed 3-fold higher activity against the *Listeria* serovar that was naturally targeted by the parental PlyPSA. However, in some cases the chimeolysin may also keep the activity of the parental enzyme that provided the CD, thus resulting in an expansion of the lytic spectrum. This was observed when the endopeptidase CD of the endolysin of the streptococcal prophage λSA2 was fused to the SH3b-type CWBD of either LysK or lysostaphin. LysK is the endolysin of the staphylococcal phage K [66], and this enzyme is known for its strong staphylolytic activity. Lysostaphin is a potent anti-staphylococcal bacteriolysin (exolysin) produced by *Staphylococcus simulans*, which targets the pentaglycine interpeptide bridge of the *S. aureus* CW ([45] and references therein). The endopeptidase-SH3b fusions (λSA2-E-Lyso-SH3b and λSA2-E-LysK-SH3b) exhibited a ~5-fold increase in staphylolytic activity when compared to the parental λSA2 endolysin, while retaining significant streptolytic activity [67]. Although not as effective as lysostaphin, the fusions were bactericidal against *S. aureus* mastitis isolates in processed cow milk and in a mouse model of mastitis [68].

Several chimeric lytic enzymes with improved enzybiotic properties have been obtained by the aleatory exchange of PLE functional modules, normally coupled to high throughput screening methods. Yang et al. [69] developed a strategy to rapidly screen a chimeolysin library containing combinations of different CDs (7 donors) and CWBDs (3 donors). One of the chimeolysins, ClyR, which was particularly active against *S. dysgalactiae* and very stable under different storage conditions, was composed of the glycosidase CD of the endolysin PlyC fused to PlySb, the CWBD of the endolysin PlySs2 (from an *S. suis* prophage). Probably because of the wide binding capacity of PlySb, ClyR showed broader lytic spectrum than PlyC, being active against several streptococcal species (including *S. pneumoniae*), *E. faecalis*, and *S. aureus*. ClyR exhibited also higher lytic activity than other known streptococcal endolysins and was capable of killing mastitis-causing streptococci in pasteurized milk. Moreover, the chimeolysin protected mice from lethal *S. agalactiae* systemic infection and exhibited antibiofilm activity towards *S. mutans* in a murine model of dental colonization [69,70].

In contrast to the previous example, the design of some chimeolysins has been rationalized based on previously known features of PLE functional domains. Cpl-711 was constructed by combining the greater affinity of the CW binding module of the pneumococcal endolysin Cpl-1 with the highly active muramidase CD of another pneumococcal endolysin, Cpl-7. The CD source for Cpl-711 construction was in fact Cpl-7S, an artificial variant of Cpl-7 with modifications introduced in its CWBD (see Section 5.2) but carrying an unchanged CD. Compared to the parental endolysins, Cpl-711 exhibited enhanced killing and antibiofilm activity in vitro and superior protection in a mouse model of pneumococcal bacteraemia (compared to Cpl-1) [71]. When this study was published, Cpl-711 was considered the most lethal anti-pneumococcal lytic enzyme with muramidase activity. Using an analogous rational, the very active CD of Cpl-7 was retargeted to the zoonotic pathogen *S. suis* through its fusion with the CWBD of LysMP, the endolysin of *S. suis* phage SMP. The generated chimeolysin, Csl2, displayed superior bactericidal and antibiofilm activity when compared to LysSMP. At the highest dose, Csl2 fully protected adult zebrafish from lethal *S. suis* infection [72].

In a very elegant study employing several endolysins, Low et al. [85] concluded that the enzymes capable of efficient lysis in absence of CWBD (see Section 5.2) carried CDs with intrinsic affinity to the CW. The binding capacity and lytic activity of isolated CDs seemed to be impaired as the negative net charge of their primary structure increased. In accordance to this, Blázquez et al. [73] coupled the less negatively charged CD of the pneumococcal endolysin Pal with the high affinity CWBD of LytA, the major pneumococcal autolysin. The resulting chimera, PL3, killed pneumococci in vitro more efficiently than the parental enzymes, while being also bactericidal against other choline-containing streptococci. In addition, PL3 showed remarkable stability at 37 °C, it resisted lyophilisation and it fully protected zebrafish embryos from *S. pneumoniae* deadly infection. PL3 conferred higher protection than other pneumococcal endolysins in this infection model.

VALs have also been used to generate chimeric lytic enzymes by functioning as an alternative source of CDs. Compared to those derived from endolysins, CDs from VALs may present increased thermostability [56,57,59]. Moreover, by following their native mode of action, VALs may be better prepared to promote lysis from without than endolysins, since the latter are naturally designed to attack the CW from within and after holin-mediated cell death [36,62]. A limitation, however, that may negatively impact VALs lytic performance is the fact that they usually lack CWBD. This has been solved by equipping VALs, or their isolated CDs, with CWBDs of heterologous origin. In fact, Rodríguez-Rubio et al. [74] demonstrated that just by adding the SH3b-type CWBD of lysostaphin to the C-terminus of HydH5, the very thermostable VAL of *S. aureus* phage phiIPLA88, was sufficient to provoke a marked increase of the staphylolytic activity of the virion lysin (chimeolysin HydH5SH3b). However, an even better chimeolysin was obtained when the same CWBD was fused to the isolated endopeptidase domain (CHAP family) of HydH5 (CD1 in Figure 3b). This chimeric lytic enzyme (CHAPSH3b) paralleled the very potent staphylolytic activity of lysostaphin in several assays. CHAPSH3b showed also the capacity to disrupt and inhibit the formation of *S. aureus* biofilms [76]. Of note, this chimeolysin proved very efficacious in eradicating *S. aureus* present in raw and pasteurized milk [75]. Interestingly, another CHAPSH3b-like chimera recently emerged from a screening of a library of 170 recombinant lytic enzymes that aimed at finding constructs with high killing activity against *S. aureus* in milk. In this case, the CHAP CD was from the endolysin LysK [86].

Another well-studied chimeolysin derived from a VAL is P128, which was built by fusing the putative endopeptidase CD of the tail-associated muralytic enzyme (TAME or Orf56) of phage K to the CWBD of lysostaphin [58]. The presence of this CWBD resulted in more than 100-fold higher bactericidal activity when compared to the isolated CD. P128 could rapidly lyse several staphylococcal species, including a representative panel of typed methicillin-resistant *S. aureus* (MRSA). A P128 concentration of 10 µg/mL was sufficient to reduce the cell counts of *S. aureus* clinical strains between 2 to 4 orders of magnitude (initially at 10^8^ CFU/mL). Within the same concentration range, P128 was also efficient at eradicating *S. aureus* biofilms and exhibited much greater thermostability than lysostaphin [77,78].

The putative CHAP-type endopeptidase CD of Ply187 (CD1 in Figure 3b) has been used to generate several chimeolysins through its combination with different CWBDs. It should be noted that Ply187 was initially described as the endolysin of the *S. aureus* phage 187 [87]. Although still referred to as endolysin in some literature, Ply187 is most probably a VAL. In fact, it shares CD organization and significant amino acidic identity with the VALs HydH5 (see above) and gp49 (from *S. aureus* phage phi11) [59,88]. The most probable endolysin of phage 187 is Orf16, a putative 251 aa peptidase lying downstream the putative holin Orf63 [89]. Addition of the SH3b-type CWBD of LysK to the CHAP CD of Ply187 resulted in a 10-fold increment of specific activity, making the chimeolysin, Ply187AN-KSH3b, more potent than LysK in some in vitro assays. As described for some of the chimeric enzymes abovementioned, Ply187AN-KSH3b was also active in milk and exhibited antibiofilm activity [79,80]. Analogous chimeolysins sharing the Ply187 CHAP domain but harboring distinct CWDBs include ClyH, Ply187AN-V12C, and ClyF. ClyH carries the CWBD of the staphylococcal phage phiNM3 endolysin. It displayed higher lytic activity than lysostaphin and than the parental lytic enzymes in vitro, it efficiently degraded MRSA biofilms, and conferred great protection to mice against systemic MRSA infection [90,91]. The V12C CWBD, from the enterococcal endolysin PlyV12, allowed expansion of the Ply187AN lytic activity to different streptococcal and enterococcal species [92]. ClyF emerged from the chimeolysin library that originated ClyR (see above), but in this case after screening for constructs showing high activity towards *S. aureus*. As for ClyR, ClyF carried the CWBD PlySb of the streptococcal endolysin PlySs2, but its lytic spectrum was limited to staphylococcal species (planktonic and biofilm growth). ClyF demonstrated superior staphylolytic activity in different in vitro environments and in murine models of bacteremia and burn wound infection [93]. Moreover, PlySb conferred enhanced thermostability and pH tolerance to the chimeolysin when compared to the isolated Ply187 CHAP domain [93].

Building on the idea that VAL CDs may be better adapted to induce lysis of actively growing bacteria, Proença et al. [62] noticed that several VALs and a few bacteriolysins (like lysostaphin and enterolysin A) shared a CD that is very unusual in endolysins (specifically, the endopeptidase domain of the M23 family). One putative VAL carrying such domain is Orf73 from the *E. faecalis* phage F170/08 (CD1 in Figure 3b). Fusion of the multimerization-prone, high-affinity CWBD of the cognate endolysin Lys170 [54] to the M23 CD of Orf73 generated the chimeolysin EC300 (holoenzyme with ~70 kDa) [62]. Both Lys170 and EC300 could efficiently lyse logarithmic phase *E. faecalis* cells resuspended in a nutrient-depleted buffered solution. However, when the lytic agents were added directly to cells growing in a rich culture medium, growth inhibition and culture lysis was only observed with EC300. The chimeolysin efficacy under growth-promoting conditions was verified for a panel of multidrug-resistant strains, including vancomycin-resistant *E. faecalis* (VRE). In these conditions, the endolysin could only elicit cell lysis if cultures were concomitantly treated with a membrane pmf-dissipating drug. Such inverse correlation between highly energized cells and their susceptibility to endolysins was later observed for other endolysins [36].

Finally, although not very frequent, augmented lytic activity and expanded antibacterial spectrum can be achieved by fusing PLEs (or their CDs) to other full-length lytic enzymes. This was the basis for the generation of the chimeric enzymes B30-443-Lyso and B30-182-Lyso, which combined the *S. agalactiae* phage B30 endolysin, or its endopeptidase domain, respectively, with mature lysostaphin. While the native endolysin lytic range was confined to *Streptococcus* species, the fusions were also active against *S. aureus*. Although with some impact in their lytic performance, the fusions could still lyse *S. agalactiae* and *S. aureus* pathogens in whey [94]. Note that B30-443-Lyso is a triple-CD fusion: two from B30 (CHAP peptidase and muramidase) and one from lysostaphin (M23 peptidase). As we will see in Section 5.4, the emergence of bacterial resistance against enzybiotics with multiple CDs seems to occur at very low levels.

### 5.2. Other Enginnering Approaches to Expand the Lytic Spectrum and Activity of PLEs

Several alternative strategies to the swapping of functional domains have been followed to obtain PLEs derivatives with improved features (Table 2). After the generation of chimeric lytic enzymes, perhaps the next most frequently used engineering approach applied to PLEs involves domain deletions. It could be expected that any deletion affecting PLE domains directly involved in peptidoglycan binding or cleavage would produce a negative impact on lytic activity. Although this was proven true when the CWBD was removed from certain endolysins targeting *L. monocytogenes*, *B. anthracis*, *S. aureus*, and *S. suis* [95,96,97,98], in other cases elimination of one CD (in dual-CD PLEs) and/or CWBDs proved to be either innocuous or to benefit PLE properties. Deletion of the binding domain was reported to produce no major effect on the in vitro lytic activity of the clostridial endolysin CS74L [99], of the streptococcal endolysin B30 [94], and of the staphylococcal endolysin LysK [100]. In fact, the two latter examples correspond to dual-CD endolysins in which the first lytic domain is an endopeptidase and the second a muramidase (B30) or an amidase (LysK). Strikingly, these two endolysins seemed also to support elimination of the second CD along with the CWBD [94,100,101]. The truncated LysK composed exclusively of the N-terminal endopeptidase domain (C165 or CHAP_k_) was reported to have enhanced activity [100], including against *S. aureus* biofilms [102], and the capacity to eliminate this bacterium from the nares of mice [103]. However, as highlighted by Becker et al. [97], this type of deletion analysis should be taken with care, as discrepant results may arise depending on the methods used to measure lytic activity. This is well illustrated by the LysK dependence on its CWBD for lytic activity, which seems to vary according to the assay conditions [104] More consistent results were obtained concerning the poor contribution of the amidase CD for the in vitro activity of LysK-like endolysins [105,106,107], and in some case its deletion resulted even in heightened lytic activity [97].

As mentioned above, some endolysin CDs may dispense the presence of a CWBD for cutting efficiently the CW murein, especially if they display a positive net charge [85]. Removal of the CWBD may actually expand the lytic spectrum and activity of endolysins, as shown for the truncated derivatives PlyL^CAT^ and PlyBa04^CAT^ that are lytic against different *Bacillus* species [85,108]. Full-length PlyL only lysed efficiently *B. cereus* and *B. megaterium*, whereas full-length PlyBa04 could only provoke lysis of *B. anthracis* and *B. cereus*. The reasons for the increment of lytic active following CWBD deletion are not fully understood, but probably size reduction may facilitate the truncated lytic enzymes in cutting through the peptidoglycan mesh [85].

On the other hand, expansion of the lytic spectrum to other bacterial species might be explained if, in the native enzymes, the CD is inhibited by intramolecular interactions with the CWBD. In this case, relief of this inhibition would require CWBD binding to the cognate CW [108,118].

Elimination of the endolysin CWBD may result in increased activity without changing significantly the lytic spectrum, indicating that specificity to certain bacteria may be an intrinsic feature of some CDs. This was observed for the clostridial endolysin CD27L and its truncation product CD27L_1-179_, which essentially corresponded to the amidase CD of the enzyme. Although exhibiting enhanced lytic activity, CD27L_1-179_ basically lysed the same range of bacteria as CD27L, except for two *Listeria* species that could only be lysed by the truncated mutant. Interestingly, the substitution of the conserved residue Leu 98 of the enzyme’s CD by its equivalent present in the listerial endolysin PlyPSA (a tryptophan residue) resulted in L98WCD27L_1-179_, which presented a cumulative augmentation in lysis efficiency towards certain *L. monocytogenes* serovars [109]. Similar results were reported for the truncation product PlyCD_1-174_ of the clostridial endolysin PlyCD [119].

Cheng and Fischetti [110] subjected PlyGBS, a streptococcal endolysin analogous to B30 (see above), to a random mutagenesis protocol for the isolation of mutants with increased lytic activity against group B streptococci (GBS). A frameshift mutation produced a truncated mutant, PlyGBS90-1, which essentially corresponded to the N-terminal endopeptidase CD (CHAP family). Besides retaining the original lytic spectrum, PlyGBS90-1 exhibited a ~28-fold increase in specific activity when compared to native PlyGBS and also improved decolonization activity in a mouse vaginal model. A similar mutant, PlyGBS94, which resulted from directed elimination of the central muramidase CD and of the putative C-terminal CWBD, gave similar results in vitro [110]. Removal of the low-activity, C-terminal glycosidase CD of the endolysin λSA2 resulted also in increased streptolytic activity, as long as the 2 central CWB motifs were preserved attached to the N-terminal endopeptidase CD (λSa2-ECC truncated product) [111].

Increasing the affinity of PLEs to target cells, by modifying CDs and/or CWBDs, in principle should result in improved lytic efficacy. Schmelcher et al. [65] showed that the listerial endolysin Ply500 equipped with an extra copy of its natural CWBD enhanced affinity by ~50-fold, which translated into greater capacity of the endolysin to act at high salt concentrations.

Despite its very active CD, the native endolysin Cpl-7 presents much lower bacteriolytic activity when compared to other pneumococcal enzybiotics, such as the endolysin Cpl-1 [113]. Díez-Martínez et al. [113] noticed that a major difference between these two lytic enzymes related to their net charge, which was much more negative in Cpl-7 at neutral pH. Negatively charged residues were particularly concentrated in the Cpl-7 CWBD, which is composed of three tandem repeats of the CW_7 binding motif. Inspired again by the work of Low et al. [85], the authors hypothesized that inverting the charge of the binding module could result in increased lytic activity. Five carefully selected amino acid substitutions were introduced per repeat, changing the net charge of the whole CWBD from −14.93 to +3.0. Overall, the mutagenized endolysin, Cpl-7S, demonstrated improved killing capacity against pneumococcal and non-pneumococcal species when compared to the wild-type Cpl-7, paralleling the Cpl-1 bactericidal activity on some pneumococcal stains (note that Cpl-1 is specific to pneumococci). A single dose of Cpl-7S was very effective in protecting from death zebrafish embryos infected with *S. pneumoniae* or *S. pyogenes* [113].

Enhanced lysis/killing of bacteria has also been described as the result of a synergistic effect when combining lytic agents displaying different enzymatic specificities, or when combining CW degrading enzymes (native or engineered) with conventional antibiotics or antimicrobial peptides. The reasons for this synergy are still poorly understood, particularly those resulting from the simultaneous action of lytic agents and antibiotics. It is conceivable that the CW peptidoglycan is more efficiently destroyed when different bonds of its structure are attacked at the same time, an effect that may also be potentiated by the facilitated access of the lytic enzymes to their substrate in these conditions. Most antimicrobial peptides act by damaging the bacterial CM, often accompanied by pmf collapse [120], and, as explained above, this condition has been shown to greatly enhance the lytic action of endolysins. Discussing the strategies relying on the simultaneous use of different antibacterial agents is out of the scope of this review. Nevertheless, they show great promise as they may translate into resensitization of bacteria to current antibiotics and into much lower doses of the individual agents being required in treatments (synergy studies summarized in [23,121]).

### 5.3. Improving the Production, Solubility, and Stability of PLEs

The engineering strategies used to enhance activity and widen the lytic spectrum of PLEs have also proved useful for ameliorating the heterologous production, solubility, or stability of some potential enzybiotics. A problem that was commonly reported for natural *S. aureus* phage endolysins was their poor solubility when heterologously overproduced. Recent optimizations of protein production and purification conditions allowed overcoming the insolubility problem at least partially. In some cases, however, chimeolysin engineering was followed to obtain highly soluble staphylolytic enzymes. One of the first reported examples was ClyS, a chimeric enzybiotic composed of the endopeptidase CD of PlyTW, the endolysin of *S. aureus* phage Twort, which was fused to the CWBD of the *S. aureus* phage phiNM3 endolysin [81]. In addition to high solubility, probably conferred by the very soluble CWBD, ClyS had a broad lytic spectrum among *Staphylococcus* species and demonstrated efficacy in murine models of MRSA colonization or infection [81,82]. By following an inverse approach, Fernandes et al. [83] successfully produced two chimeolysins by fusing the highly soluble CDs of two different *E. faecalis* endolysins (Lys168 and Lys170) to the CWBD of Lys87, a broadly active staphylococcal endolysin with solubility issues. Remarkably, the resulting chimeras Lys168-87 and Lys170-87 showed not only to be broadly active against a large cohort of *S. aureus* isolates, which included representatives of the most relevant MRSA pandemic clones, but also against other staphylococcal, streptococcal, and enterococcal species.

Engineered chimeolysins may also yield enzybiotics that are more thermostable and therefore that may better preserve activity during storage. Besides the cases of high thermostability (or good stability under different storage conditions) mentioned in the previous sections (see ClyR, PL3, CHAPSH3b, P128, and ClyF), other cases of heat-stable enzybiotics have been described as result of chimeolysin engineering. For example, with the goal of producing a stable enzybiotic to combat *C. perfringens* in poultry, Swift et al. [84] assembled the amidase CD of the thermostable endolysin PlyGVE2 (from *Geobacillus* phage ФGVE2) with the CWBD of PlyCP26F (endolysin of *C. perfringens* phage ФCP26F). The produced chimera, PlyGVE2CpCWB, preserved more than 57% of its activity after 30 min at 55 °C, whereas the parental endolysin PlyCP26F was completely inactivated by this treatment.

The presently available bioinformatics tools may also be of great help for optimization of protein heterologous expression. A detailed in silico analysis of the primary, secondary, and tertiary structures of the staphylococcal endolysin LysK allowed precise sequence delimitation of the enzyme’s CHAP peptidase and amidase CDs, and elimination of putative protein segments contributing to instability/insolubility [117]. The *E. coli* codon-optimized coding sequences of the two CDs were then connected by an artificial linker (GSH_6_GS), which contained the hexahistidine tag for protein purification by affinity chromatography. Moreover, the CHAP-Amidase fusion was N-terminally fused to the signal peptide sequence of PelB, which allowed targeting of the enzybiotic to the *E. coli* periplasm during overproduction. This strategy resulted in high production of a soluble (~12 mg soluble protein per liter of culture), stable, and highly active enzybiotic, with a lytic spectrum covering *S. aureus*, *S. epidermidis*, *E. faecalis*, and *E. faecium* [117].

As described above, the multimeric endolysin PlyC is composed of two subunits, the dual-CD PlyCA and PlyCB, with the latter forming the octameric, ring-shaped CWBD. PlyC loses activity rapidly at 45 °C, which may hint for a reduced shelf life. Interestingly, the poor thermal stability of PlyC is conferred by the CD subunit, since PlyCB resists up to ~90 °C [112]. PlyCA was subjected to a random mutagenesis method based on error-prone PCR, followed by a screening for mutants with enhanced thermostability. One of the selected variants, 29C3, showed more than 2-fold increase in kinetic stability at 45 °C. This translated into better preservation of activity than the native endolysin when tested at different temperatures and incubation periods [112]. In a subsequent study, the same authors performed a computational modelling study to find PlyCA residues that, upon change, would likely result in a Δ*G* decrease (stabilizing mutations). One of the mutants, PlyC (PlyCA) T406R, was confirmed experimentally to have a denaturation temperature increased by ~2.2 °C and a kinetic stability augmented 16-fold over the wild type PlyC [114].

Many enzybiotics are expected to have a relatively short half-life after reaching the blood stream, since proteins below 45–50 kDa tend to be rapidly cleared from plasma by renal filtration [122]. Aiming at extending the half-life of the endolysin Cpl-1, Resch et al. [115] engineered a dimeric version (Cpl-1^C45S,D324C^) of the lytic enzyme by eliminating and introducing appropriate Cys residues in the primary sequence. Dimerization occurred through intermolecular disulphide bonding involving the Cys residue inserted at position 324, yielding a 74 kDa enzybiotic. The Cpl-1 dimer showed a ~10-fold decrease in plasma clearance (in mice) compared to native Cpl-1, while doubling the specific activity of Cpl-1. Conjugation with non-immunogenic polymers like polyethylene glycol (PEGylation) or polycationic polymers such as poly-l-lysines has proved successful in extending the half-life of many biological molecules, including the lytic enzyme lysostaphin, and in decreasing immunogenicity, proteolysis, and instability [123,124,125]. PEGylation of Cpl-1, however, resulted in abolishment of the endolysin lytic activity in vitro [126], suggesting some limitations in the application of this strategy with this kind of enzymes. Currently known multimeric PLEs (native or engineered, see above) have a molecular weight above the renal filtration cut-off, meaning that they may be available in circulation for longer time.

### 5.4. Minimizing Development of Resistance to PLEs

The presently available studies support that emergence of resistance to endolysins should occur at much reduced levels when compared to antibiotics and other lytic enzymes like exolysins (studies summarized in [15,127]). It has been hypothesized that endolysins probably evolved to target essential CW components that cannot be easily modified without seriously compromising bacterial fitness, thus ensuring virion release and phage survival at the end of infection [127]. Nevertheless, in a recent study Becker et al. [116] hypothesized that the chances of resistance development could be further reduced if multiple, different CDs were assembled in a single enzybiotic. This was tested using fusions incorporating the two CDs of LysK and the CD of lysostaphin, in two possible configurations, K-L or L-K. In K-L, a LysK segment encompassing its two CDs was fused to the N-terminus of lysostaphin. In the L-K configuration, the same dual-CD segment was inserted between the CD and the SH3b-type CWBD of lysostaphin. The chimeolysins carried thus the endopeptidase (CHAP) and amidase CDs of LysK and the endopeptidase CD (M23) of lysostaphin, while sharing the same C-terminal CWBD (SH3b from the exolysin). Depending on the assay, the triple-CD fusions showed slightly decreased or equivalent in vitro activity when compared to the parental lytic enzymes. However, when the *S. aureus* strain Newman was exposed to sub-lethal doses of the four agents during 10 growth rounds, either in liquid cultures or in solid medium, the emergence of resistance (assessed as the MIC fold-increase) was much lower with the chimeolysins, especially compared to lysostaphin. The fusions were effective as the parental enzymes at eradicating biofilms, whereas the L-K fusion exhibited superior killing activity in a rat nasal model.

In a previous study, Rodríguez-Rubio et al. [128] evaluated the emergence of *S. aureus* resistance to four lytic enzymes: lysostaphin, the LysK-like endolysin LysH5 (from phage phiIPLA88), and the fusions HydH5Lyso and HydH5SH3b. The latter chimeolysins were generated by adding lysostaphin, or its SH3b CWBD, respectively, to the dual-CD VAL (HydH5) of phage phiIPLA88 (Figure 3b). While *S. aureus* cells resistant to lysostaphin were rapidly isolated after 1 or 2 growth cycles, none could be detected after exposure to the endolysin or to the chimeolysins in 10 rounds of subculturing, either in liquid or solid medium.

### 5.5. Enhancing PLEs as Antibacterials towards Gram-Negative Bacteria

As stated above, the outer membrane (OM) of Gram-negative bacteria (and also that of mycobacteria) was long seen as a major obstacle impeding access of exogenously-added PLEs to the murein layer of the CW. Interestingly, in recent years a growing number of endolysins have been described that have some intrinsic capacity to cross the OM (reviewed in [23,52]). Endolysin C-termini that are highly positively charged and/or that form amphipathic helices appear to be common requirements for traversing the Gram-negative OM. In some cases, when produced as synthetic peptides, these C-terminal segments were even shown to be bactericidal [129,130]. Nevertheless, when compared to endolysins of phages infecting Gram-positive bacteria, often much higher concentrations of these OM-crossing lytic enzymes are required for significant cell killing. Therefore, efforts have been made to find agents that could act either attached or in conjunction with PLEs to facilitate their access to the peptidoglycan (reviewed in [23,52]). Regarding protein engineering strategies, two principal types of fusions have been successfully employed to improve endolysin OM penetration (Table 3). In the first case, the lytic enzymes are fused to domains that target them to specific receptor/transport systems of the OM. The second approach relies on the fusion of endolysins to OM-destabilizing peptides, generating the so-called Artilysins^®^ [52].

In the first example of transporter-mediated crossing of the OM, Lukacik et al. [131] fused the N-terminal binding domain of pesticin, a lytic bacteriocin produced by *Yersinia pestis*, to the N-terminus of *E. coli* phage T4 lysozyme. The bacteriocin binding domain specifically targets the OM transporter FyuA, which is responsible for the uptake of the toxin. FyuA is a major virulence factor present in *Y. pestis* and some pathogenic *E. coli* strains. *Y. pestis* produces an immunity protein (Pim) that binds the muramidase CD of pesticin, thus conferring protection against its own pesticin. The authors showed that bacterial cells expressing FyuA were killed by the pesticin-T4 lysozyme hybrid, although not as effectively as with the native bacteriocin. In contrast, the same cells were unaffected by the addition of purified T4 lysozyme. Since lethality was the result of peptidoglycan degradation in the periplasm, the binding domain was considered responsible for FyuA-mediated transport of the fusion across the OM. Most importantly, the fusion could kill Pim-producing cells, because the immunity protein was unable to bind the T4 lysozyme moiety, thereby including *Y. pestis* in its spectrum of activity. An analogous approach was used to promote OM translocation of Lysep3, the endolysin of *E. coli* phage vB_EcoM-ep3. In this case, a protein segment encompassing the translocation and receptor binding domains of colicin A was fused to the N-terminus of Lysep3. In contrast to the endolysin, the colicin-Lysep3 fusion could kill *E. coli* cells, most likely because it was translocated to the periplasm by the TolB machinery after binding to the OM receptor BtuB [132].

Despite the elegance of the previous approaches, some potential limitations are foreseen. In order to act, these enzybiotics require the presence of the corresponding receptors in the OM of target cells. Although this selectivity may avoid the collateral damage to the natural microbiota (particularly in the case of pesticin-T4 lysozyme), it may also restrict the application to certain bacterial strains. Moreover, as highlighted by Briers and Lavigne [52], point mutations may easily arise in the receptors/transporters that might impair uptake of the hybrid lytic enzymes. These limitations are less likely to occur with Artilysins.

In the basic Artilysin technology, one peptide with the capacity to destabilize the negatively-charged outer leaflet of the OM, which is essentially composed of lipopolysaccharides (LPS) and some phospholipids, is fused to a given endolysin. Regarding their biochemical properties, the peptides can be polycationic, hydrophobic, or amphipathic, with some actually deriving or mimicking natural antimicrobial peptides (AMPs). Although destabilizing the OM by different mechanisms, all these peptides promote OM crossing of the endolysins without requiring the presence of dedicated cell envelope receptors/transporters [52].

In one of the first studies to produce effective Artilysins, seven different putative OM-destabilizing peptides were fused to the N-terminus of two modular endolysins, OBPgp279 and PVP-SE1gp146 (from *Pseudomonas fluorescens* and *Salmonella enterica* phages, respectively) [133]. Of the 14 possible combinations, those that mostly increased the bactericidal activity of OBPgp279 and PVP-SE1gp146, evaluated against *P. aeruginosa*, were LoGT-001 and LoGT-008, respectively. These Artilysins carried a polycationic nonapeptide (PNCP, aa sequence KRKKRKKRK) fused to the N-terminus of the endolysins. The killing effect of both Artilysins was highly potentiated in presence of 0.5 mM EDTA, a well-known OM permeabilizer. Increasing the distance between PCNP and OBPgp27 with a 16-aa segment of alternating Ala and Gly residues (LoGT-023) further improved the antibacterial effect against *P. aeruginosa* and expanded efficient killing activity to *A. baumannii*. The Artilysin with higher bactericidal activity towards *E. coli* in absence of EDTA was LoGT-037, which carried PCNP and HPP (hydrophobic pentapeptide FFVAP) tandemly fused to the N-terminus of OBPgp279. These results show the power and versatility of the Artilysin technology. One of the Artilysins, LoGT-008, proved its efficacy in rescuing human keratinocytes and *Caenorhabditis elegans* from lethal infection with the highly virulent *P. aeruginosa* strain PA14. The endolysin Lysep3 (see above) could also kill *E. coli* from without after the addition of a 15-aa polycationic peptide to its C-terminus [138].

Art-175 is an example of an Artilysin that was generated by fusing a natural AMP to an endolysin. Specifically, the broad-spectrum sheep myeloid antimicrobial peptide 29 (SMAP-29), a 29-aa α-helical cationic peptide produced by sheep leukocytes, was fused to the endolysin KZ144 of *P. aeruginosa* phage φKZ [134]. Actually, Art-175 carries a mutated version of KZ144, obtained after three Cys to Ser substitutions. This modification impaired oligomerization and conferred structural stability to the Artilysin. In contrast to KZ144 that poorly killed *P. aeruginosa* (~0.5 Log reduction), Art-175 had nearly the same lethal effect as SMAP-29, with almost complete elimination of bacterial cells (>4 Log reduction). Art-175 had a very wide spectrum of activity among environmental and clinical *P. aeruginosa* isolates, including multidrug-resistant strains. Other susceptible pathogens included *Klebsiella pneumoniae* and *Salmonella enterica* serovar Enteritidis. Importantly, the lethal character of Art-175 depended on the lytic action of the KZ144 moiety and did not rely on a potential bactericidal effect of the attached SMAP-29. In fact, the Artilysin had no significant antibacterial activity against *S. aureus*, whereas the isolated SMAP-29 efficiently killed this bacterial species. As expected from the different mode of action of enzybiotics versus antibiotics, Art-175 efficiently eliminated *P. aeruginosa* persister cells, in contrast to ciprofloxacin (both tested at 10 and 30X the MIC) [134].

Remarkably, a subsequent study revealed that Art-175 also displayed potent bactericidal activity against multidrug-resistant *Acinetobacter baumannii*, outcompeting ciprofloxacin and tobramycin in time-kill assays with stationary-phase cultures [135]. As observed for *P. aeruginosa*, persister cells of *A. baumannii* were efficiently eliminated by Art-175. Curiously, the endolysin KZ144 exhibited also antipersister activity, although with about half the efficacy of the Artilysin. Of note, the *P. aeruginosa* OM lipoprotein OprI was shown to be responsible for the susceptibility of this bacterium to SMAP-29 (as well as to other AMPs), instead of the surface LPS [139]. Mutations in OprI or in the functional homologues in other bacteria could therefore impair Art-175 uptake. However, repeated exposure (20 cycles) of either *P. aeruginosa* or *A. baumannii* to subinhibitory concentrations of Art-175 did not lead to resistance development other than a 2-fold increase of the MIC of some strains. In contrast, similar experiments with ciprofloxacin led to the rapid isolation of highly resistant bacteria [134,135]. Quite recently, Art-175 was also shown to be bactericidal against colistin-resistant, *mcr*-*1*-positive *E. coli* isolates. This shows that the modifications of the lipid A moiety of LPS responsible for colistin resistance have no impact on the antibacterial activity of Art-175 [136].

One of the endolysins (OBPgp279) that was used to generate the LoGT Artilysin series (see above) was also modified with an AMP-derived peptide, in this case the first eight amino acid residues of cecropin A, which is produced by the cecropia moth [140]. The modified endolysin (PlyA) demonstrated efficient and wide bactericidal activity in vitro against several clinical isolates of *A. baumannii* and *P. aeruginosa*, with the former species being in general more susceptible. However, in contrast to Art-175, PlyA was poorly active against cells in stationary growth phase and required the OM permeabilizer EDTA for effective killing.

As explained above, several endolysins from Gram-negative systems with intrinsic capacity to traverse the OM seem to depend on positively charged and/or amphipathic C-termini for crossing this cell barrier. Interestingly, at least in one case OM-crossing properties were also attributed to the C-terminus of an endolysin naturally designed to act on a Gram-positive bacterium, the endolysin of *Bacillus amyloliquefaciens* phage Morita2001 ([141] and references therein). The Morita2001 endolysin, which is 98% identical to that of the *B. subtilis* phage φ29, is a typical Gram-positive endolysin with an N-terminal lysozyme CD linked to a C-terminal CWBD made of two tandem LysM motifs [38]. In this lytic enzyme, the LysM motifs are rich in positive (mostly lysine) and hydrophobic residues. A deletion analysis study showed that the capacity to penetrate the *P. aeruginosa* OM resided in a C-terminal region denominated D8, which basically encompassed the two LysM motifs [141]. Wang et al. [137] envisaged that D8 could be used to promote the OM translocation of other endolysins. This was tested by fusing D8 to the C-terminus of the endolysin Lysep3 (see above). Differently from the isolated Lysep3 and D8, the fusion Lysep3-D8 was bacteriostatic against several *E. coli* clinical isolates, a reduced number of *P. aeruginosa* and *A. baumannii* strains, and even one *Streptococcus* sp. The bactericidal effect of Lysep3-D8 was confirmed, being particularly obvious for 5 out of 14 *E. coli* isolates (enzybiotic at 100 µg/mL).

In quite an innovative approach, Rodríguez-Rubio et al. [142] decided to test whether the OM-destabilizing peptides used in “Artilysation” of endolysins from Gram-negative systems could also somehow enhance the activity of lytic enzymes directed to Gram-positive bacteria. In this work, the polycationic nonapeptide (PCNP) used to construct the LoGT Artilysin series described above was added to the C-terminus of the streptococcal endolysin λSa2lys (from *S. agalactiae* prophage λSA2). The resulting Artilysin, Art-240, retained the broad anti-streptococcal spectrum of the parental endolysin, but its bactericidal activity was enhanced (killing rate ~2-fold higher). For example, a 2 Log reduction in cellular counts required a 12-fold higher dose of λSa2lys. The superior killing activity of Art-240 was observed over a wide range of pH values and salt concentrations. The authors speculated that the improved killing performance of Art-240 probably resulted from an increased affinity to the streptococcal CW, conferred by the positive amino acid residues of PCNP, since the cell surface is negatively charged due to the presence of anionic teichoic acids.

### 5.6. Targeting Intracellular Bacteria with PLEs

The use of enzybiotics has been mainly foreseen to combat bacterial pathogens that mostly present an extracellular life style when infecting or colonizing animal hosts, as happens with the species mentioned throughout this review. However, many of these pathogens (e.g., *S. aureus*, *S. pneumoniae*, *S. pyogenes*, *E. coli*) can also have a phase of intracellular inhabitance that can be important for the establishment of infection, to evade antimicrobials and the immune system, or to persist within the host [143]. Somewhat unexpectedly, recent studies have uncovered the capacity of enzybiotics to get inside mammalian cells and kill resident bacteria. The first work used LysK (K), lysostaphin (L), and their fusion derivatives K-L and L-K. These fusions are triple-CD chimeolysins that were described to significantly reduce the chances of emergence of resistant bacteria (see Section 5.4) [116]. The authors assumed that these lytic agents could not enter animal cells. Therefore, following a previous proposal [144], eleven protein transduction domains (PTDs) were individually fused to the C-terminus of the four enzybiotics. PTDs are typically short, highly cationic peptides, some naturally occurring, which promote protein transduction across eukaryotic cell membranes [145]. Curiously, the PTDs only produced the expected effect when added to lysostaphin, with some L-PTDs capable of killing *S. aureus* internalized in three different cell lines. Strikingly, the K-L fusion had the intrinsic capacity to reduce *S. aureus* intracellular counts in two different cells lines, and addition of a PTD did not improve its killing effect. The K-L-PTD1 fusion was nevertheless superior at clearing *S. aureus* in a murine model of mastitis and in biofilm eradication when compared to K-L [116].

The results of the previous work further support that appending cationic peptides to enzybiotics designed to act on Gram-positive bacteria may enhance their antibacterial activity in certain conditions (see Art-240 above). In addition, the study brought to light the possibility of enzybiotic uptake by animal cells. In fact, quite recently Shen et al. [146] discovered that the multimeric streptococcal endolysin PlyC could significantly reduce the counts of *S. pyogenes* cells internalized in different human epithelial cell lines, including primary tonsillar epithelial cells. Two other streptococcal endolysins, B30 and Ply700, failed to significantly reduce intracellular *S. pyogenes* in the same assays. PlyC movement across the membrane of epithelial cells was shown to depend on the PlyCB subunit and on its capacity to bind membrane phospholipids, particularly phosphatidylserine. PlyC uptake depended also on caveolae-mediated endocytosis, and endolysin internalization was not harmful to epithelial cells. These results opened new venues for the enzybiotics field, as it is now attractive to explore the potential of these antibacterial agents as killers of intracellular pathogens. In addition, the identification of enzyme’s elements like PlyCB that facilitate transport across animal cell membranes provides new engineering opportunities, as these elements may be used to deliver heterologous CDs or other cargos.

## 6. Conclusions

Enzybiotics derived from phage lytic products are among the most promising alternatives to fight antibiotic-resistant bacteria. In recent years, there has been great investment in modification and engineering approaches to develop enzybiotics with high therapeutic potential to enter clinical trials. Efforts have been made to obtain products with maximized bactericidal activity in vitro and in vivo, with good coverage of the most relevant clinical strains and with the necessary features for large scale production, formulation, and storage. Among the multiple strategies that have been followed, of particular note is the engineering of chimeolysins, the modification of PLEs net charge, and the Artilysin technology. Chimeolysins are among the most potent enzybiotics produced thus far and have been improved by the recent incorporation of VAL CDs in their design. Several studies point to an increase in the affinity and activity of PLEs when their net charge is progressively shifted from negative to positive, an effect that is explained by the fact that the bacterial cell envelope is usually negatively charged. Artilysation of PLEs was a crucial advancement to make enzybiotics a credible alternative to fight Gram-negative pathogens. Interestingly, there is a good chance that this technology may be adapted to improve also enzybiotics targeting Gram-positive bacteria. Finally, the recent discovery that PLEs can cross the membrane of animal cells and kill residing bacteria opens the possibility of using these agents to target obligatory or facultative intracellular pathogens.

## Figures and Tables

**Table 1:** Examples of PLE engineering through domain shuffling and resulting improvements.

Chimeolysin	CD Source	CWBD Source	Susceptible Bacteria	In Vivo Assay(s)	Outcome	Reference
EAD118_III_CBDPSA	Endopeptidase CD of Ply118 (endolysin *L. monocytogenes* phage A118)	PlyPSA (endolysin *L. monocytogenes* phage PSA)	*L. monocytogenes*	Not reported	3-fold higher activity compared with parental PlyPSA	[65]
λSA2-E-Lyso-SH3b and λSA2-E-LysK-SH3b	Endopeptidase CD of λSA2 (endolysin *S. agalactiae* prophage λSA2)	SH3b-like CWBD of Lysostaphin or of LysK (endolysin of *S. aureus* phage K)	Staphylococci, streptococci	Mouse model of mastitis	Efficient activity extended to *S. aureus* while retaining significant streptolytic activity	[67,68]
ClyR	Glycosidase CD (first 153 aa of PlyCA subunit) of PlyC (endolysin streptococcal phage C_1_)	PlySs2 (endolysin *S. suis* prophage)	Several streptococcal species (including *S. pneumoniae*), *E. faecalis*, *S. aureus*	Murine models of *S. agalactiae* systemic infection and of *S. mutans* dental colonization	Higher activity and broader lytic spectrum than the parental and other streptococcal endolysins. Stable under storage	[69,70]
Cpl-711	Muramidase CD of Cpl-7S (improved variant of pneumococcal endolysin Cpl-7, see below)	Cpl-1 (endolysin pneumococcal phage Cpl-1)	*S. pneumoniae*, *S. mitis*	Murine bacteraemia model	Greater killing and antibiofilm activity than parental endolysins in vitro. Superior protection compared with Cpl-1 in a mouse model of bacteraemia	[71]
Csl2	Muramidase CD of Cpl-7 (endolysin pneumococcal phage Cp-7)	LySMP (endolysin *S. suis* phage SMP)	*S. suis*, *S. pseudopneumonia*, *S. mitis*, *S. oralis*	Adult zebrafish model of infection	Superior bactericidal and antibiofilm activity than parental LysSMP	[72]
PL3	Amidase CD of Pal (endolysin pneumococcal phage Dp-1)	First two choline-binding repeats of Pal and the last four of LytA (major pneumococcal autolysin)	*S. pneumonia* and other choline-containing streptococci	Zebrafish embryo infection model	Superior bactericidal activity than parental enzymes and high stability	[73]
CHAPSH3b	Endopeptidase CD (CHAP) of HydH5 (VAL *S. aureus* phage phiIPLA88)	SH3b-like CWBD of lysostaphin (bacteriolysin *S. simulans*)	*S. aureus*, *S. epidermidis*	Not reported	Thermostability. Much higher activity than the parental HydH5	[59,74,75,76]
P128	Putative endopeptidase (CHAP) of Orf56 (VAL *S. aureus* phage K)	SH3b-like CWBD of lysostaphin (bacteriolysin *S. simulans*)	*S. aureus*, *S. epidermidis*, *S. carnosus*, *S. simulans*	Rat nasal colonization model (*S. aureus* USA 300)	P128 has much higher killing activity than the isolated CHAP CD of Orf56. Effective antibiofilm activity. Better thermostability than lysostaphin	[58,77,78]
Ply187AN-KSH3b	Putative endopeptidase CD of Ply187 (PLE from *S. aureus* phage 187)	SH3b-like CWBD of LysK (endolysin of *S. aureus* phage K)	*S. aureus* and other staphylococcal species	Mouse model of *S. aureus* Endophthalmitis	More active than native Ply187 and Ply187AN truncated Enzyme. Effective antibiofilm activity	[79,80]
EC300	Putative endopeptidase CD (M23) of Orf73 (putative VAL *E. faecalis* phage F170/08)	Oligomerization-prone CWBD of Lys170 (endolysin *E. faecalis* phage F170/08)	Multidrug-resistant *E. faecalis*, including VRE	Not reported	In contrast to the parental endolysin, EC300 lysis *E. faecalis* actively growing in rich medium	[62]
ClyS	Endopeptidase CD of PlyTW (endolysin *S. aureus* phage Twort)	Endolysin *S. aureus* phage phiNM3 (highly soluble CWBD not related to the very common SH3b)	*S. aureus*, *S. sciuri*, *S. simulans*, *S. epidermidis*	Different murine colonization/infection models (nasal, skin and systemic)	Broad-spectrum activity and high solubility when compared to most staphylococcal endolysins	[81,82]
Lys168-87	Putative endopeptidase CD of Lys168 (endolysin *E. faecalis* phage F168/08)	Putative CWBD of Lys87 (endolysin *S. aureus* phage F87s/06)	Staphylococci, *E. faecalis*, *E. faecium*, *S. pyogenes*	Not reported	High solubility compared to most native PLEs targeting *S. aureus*. Expanded spectrum of activity	[83]
PlyGVE2CpCWB	Amidase CD of PlyGVE2 (endolysin *Geobacillus* phage ФGVE2)	PlyCP26F (endolysin *C. perfringens* phage ФCP26F)	*C. perfringens*	Not reported	Better thermostability than parental PlyCP26F	[84]

**Table 2:** Other examples of PLE engineering strategies and major outcomes.

Engineering Approach	Example(s) ^1^	Engineering Details ^1^	Susceptible Bacteria	In Vivo Assay(s)	Outcome	Reference
Fusion to lytic enzymes	B30-443-Lyso B30-182-Lyso	Fusion of *S. agalactiae* phage B30 endolysin (or of its endopeptidase CD) to *S. simulans* lysostaphin	Several streptococcal species, including pathogens and dairy bacteria. *S. aureus*	Not reported	Lytic spectrum extended to *S. aureus* and increased activity (B30-182-Lyso)	[94]
Domain deletion	CHAP_K_	CHAP_K_ corresponds to the endopeptidase (CHAP) CD of LysK (first 165 aa of de endolysin of *S. aureus* phage K)	*S. aureus*	*S aureus* elimination in the nares of mice	Higher lytic activity than LysK	[100,102,103]
	PlyL^CAT^ (amidase) PlyBa04^CAT^ (muramidase)	Deletion of the C-ter CWBDs of PlyL and PlyBa04, the endolysins of *B. anthracis* λBa02 Prophage and *B. anthracis* phage Ba04, respectively	*B. cereus*, *B. megaterium*, *B. anthracis*, *B. subtilis*	Not reported	Extended lytic spectrum. Enhanced lytic activity (especially against *B. subtilis* in the case of PlyL^CAT^)	[85,108]
	CD27L_1-179_ (N-ter amidase CD)	Deletion of the C-ter CWBD of the clostridial endolysin CD27L	*Clostridium* spp. (including *C. difficile*), *Bacillus* spp., *Listeria* spp.	Not reported	Increased lytic activity and spectrum extended to two additional *Listeria* sp.	[109]
	PlyGBS94	PlyGBS94 corresponds to the first 146 aa of native PlyGBS (endolysin *S. agalactiae* phage NCTC 11261), carrying only a endopeptidase CD	Group B streptococci (*Streptococcus agalactiae*)	Not reported	~25-fold increase of specific activity	[110]
	λSa2-ECC	Deletion of C-ter glycosidase CD of λSA2 endolysin (*S. agalactiae* prophage λSA2)	Several streptococcal species and few *S. aureus* strains	Not reported	Increased activity towards certain streptococcal strains and few *S. aureus* strains	[111]
Domain addition	HydH5SH3b	Addition of lysostaphin CWBD (SH3b) to VAL HydH5 of *S. aureus* phage phiIPLA88	*S. aureus*, *S. epidermidis*	Not reported	Higher activity than the parental HydH5	[74]
Domain duplication	EAD_CBD500-500	Extra copy of CWBD added to Ply500 (endolysin *L. monocytogenes* phage A500)	Essentially *Listeria* spp.	Not reported	Much higher affinity improves endolysin activity at high salt concentrations	[65]
Random mutagenesis	PlyGBS90-1	Frameshift mutation truncates PlyGBS at aa 141 and adds 13 aa	Group B streptococci (*Streptococcus agalactiae*)	Decolonization in a mouse vaginal model	~28-fold increase of specific activity, although less stable than native PlyGBS in certain conditions. Improved killing activity in vivo	[110]
	29C3 mutant of PlyC	Mutation-prone PCR of PlyCA subunit of PlyC (endolysin streptococcal phage C_1_)	*S. pyogenes*	Not reported	The 29C3 mutant exhibits higher thermostability than PlyC, which should translate into extended shelf life	[112]
Site-directed mutagenesis	Cpl-7S	15 aa substitutions added positive charges to the CWBD of pneumococcal endolysin Cpl-7 (from −14.93 to +3.0 at neutral pH)	*S. pneumoniae*, *E. faecalis*, *S. mitis*, *S. pyogenes*, and, to a lesser extent, *S. dysgalactiae* and *S. iniae*. *E. coli* and *P. putida* in presence of carvacrol	Zebrafish embryo infection model (*S. pneumoniae* and *S. pyogenes*)	Improved killing activity compared to the native Cpl-7 endolysin	[113]
	(PlyC)T406R	T406R substitution in PlyCA subunit of PlyC (endolysin streptococcal phage C_1_)	*S. pyogenes*	Not reported	Thermostabilization of PlyC (16-fold increase of half-life at 45 °C), although with moderate loss of lytic activity in vitro	[114]
Multimerization	Cpl-1 dimer	Cpl-1^C45S,D324C^. Introduction of Cys residues at aa position 324 allowed intermolecular disulphide bonding. The C45S substitution avoided unwanted interactions with this Cys residue	*S. pneumoniae*	Not reported	2-fold increase of antipneumococcal activity and ~10-fold decrease in plasma clearance (mice) compared to native Cpl-1	[115]
Mixed approaches	L98WCD27L_1-179_	Deletion of CD27L C-ter CWBD and L98W mutation in CD27L CD	*Clostridium* spp. (including *C. difficile*), *Bacillus* spp., *Listeria* spp.	Not reported	The L98W mutation further increased lytic activity of CD27L_1-179_ against *L. monocytogenes*	[109]
	K-L K-L-PTD L-K L-K-PTD (triple-CD PLEs, i.e., 3 distinct CDs)	LysK/Lysostaphin chimeras added or not of protein transduction domains (PTD). K-L: CHAP-Amidase CDs of LysK fused to lysostaphin. L-K: LysK CDs inserted between the CD (M23) and CWBD (SH3b) of lysostaphin	*S. aureus* and coagulase negative staphylococci	Decolonization in rat nasal model. Murine model of mastitis	The presence of 3 distinct CDs in the chimeras reduces emergence of resistant strains. Superior killing activity of L-K in rat nasal model	[116]
	CHAP-Amidase	Codon-optimized CHAP and amidase CDs of LysK (endolysin *S. aureus* phage K) connected by the linker GSH_6_GS. No CWBD	*S. aureus*, S. *epidermidis*, *E. faecium*, and *E. faecalis*	Not reported	Enhanced production, stability, and solubility by improving codon-usage and the properties of primary, secondary, and tertiary structures	[117]

^1^ N-ter: N-terminal; C-ter: C-terminal.

**Table 3:** Fusion of PLEs with domains or peptides that promote crossing of the OM barrier.

Engineering Approach	Example(s)	Engineering Details ^1^	Susceptible Bacteria	In Vivo Assay(s)	Outcome	Reference
Fusion to domains targeting OM receptor/transport systems	Pesticin-T4 lysozyme hybrid	Pesticin (bacteriocin) domain targeting FyuA (OMP) fused to the N-ter of *E. coli* phage T4 endolysin	FyuA-expressing pathogenic bacteria (*Y. pestis*, *Y. pseudotuberculosis*, uropathogenic *E. coli*)	-	The hybrid protein crosses the OM through FyuA-mediated transport	[131]
Fusion to domains or peptides that destabilize the OM	LoGT-001 LoGT-008	LoGT-001: PCNP (polycationic nonapeptide) connected to the N-ter of OBPgp279 (endolysin *P. fluorescens* phage OBP) LoGT-008: PCNP connected to the N-ter of PVP-SE1gp146 (endolysin *S. enterica* phage PVP-SE1)	*P. aeruginosa.* Other Artilysins of the LoGT series also killed effectively *A. baumannii* and *E. coli* (≥1 Log reduction). Killing of *S.* *Typhimurium* required EDTA	*C. elegans* infection assay (LoGT-008)	The PCNP tag increased the intrinsic antibacterial of two modular endolysins (OBPgp279 and PVPSE1gp146) by facilitation OM crossing	[133]
	Art-175	Antimicrobial peptide SMAP-29 fused to the N-ter of mutated KZ144 (endolysin *P. aeruginosa* phage φKZ)	*P. aeruginosa* (and few other *Pseudomonas* spp.), *K. pneumoniae*, *A. baumannii*, colistin-resistant *E. coli*	-	In contrast to KZ144, Art-175 crosses the outer membrane and efficiently kills target cells. Capacity to eliminate *P. aeruginosa* and *A. baumannii* persister cells. Art-175 outcompetes conventional antibiotics in bactericidal activity against *A. baumannii*	[134,135,136]
	Lysep3-D8	Lysep3 (endolysin *E. coli* phage vB_EcoM-ep3) fused to region D8 of the endolysin of *B. amyloliquefaciens* phage Morita2001	*E. coli*, *P. aeruginosa* (3 strains), *A. baumannii* (1 strain), *Streptococcus* sp. (1 strain)	-	In contrast to isolated Lyse3 and D8, Lysep3-D8 has bactericidal activity	[137]

^1^ N-ter: N-terminus.
