# Correction: São-José, C. Engineering of Phage-Derived Lytic Enzymes: Improving Their Potential as Antimicrobials. *Antibiotics* 2018, *7*, 29

**DOI:** 10.3390/antibiotics7030056

**Published:** 2018-07-04

**Authors:** Carlos São-José

**Affiliations:** Research Institute for Medicines (iMed.ULisboa), Faculty of Pharmacy, Universidade de Lisboa, Av. Prof. Gama Pinto, 1649-003 Lisboa, Portugal; csaojose@ff.ul.pt; Tel.: +351-217-946-420

The author wishes to make the following corrections to this paper, as the author has recently been made aware by Dr. Hang Yang (Wuhan Institute of Virology, Chinese Academy of Sciences, Wuhan, China) of the erroneous description of the chimeolysin ClyR in the text and Table 1 of this paper, which was recently published in *Antibiotics* [1].

## 1. Change in Main Body Paragraph

In the second paragraph of Section 5.1, a sentence regarding ClyR currently reads as follows:

“One of the chimeolysins, ClyR, which was particularly active against *S. dysgalactiae* and very stable under different storage conditions, was composed of the glycosidase CD of the endolysin PlyC fused to PlySb, the CWBD of the endolysin PlySs2 (from an *S. suis* prophage).”

To set straight the scientific record, we would like to make the following correction:

“One of the chimeolysins, ClyR, which was particularly active against *S. dysgalactiae* and very stable under different storage conditions, was composed of PlyCAC, the amidase CD of the endolysin PlyC, fused to PlySb, the CWBD of the endolysin PlySs2 (from an *S. suis* prophage).”

## 2. Change in Table 1

The text that appears in the “CD source” column regarding ClyR currently reads as follows:

“Glycosidase CD (first 153 aa of PlyCA subunit) of PlyC (endolysin streptococcal phage C1)”

To set straight the scientific record, we would like to make the following correction:

“Amidase CD (CHAP) of PlyC (endolysin streptococcal phage C1)”

The author would like to apologize for any inconvenience caused to the readers by these changes. The change does not affect the major highlights of the article. The manuscript will be updated and the original will remain online on the article webpage. 
